# Cellulose-Based Metallogels—Part 3: Multifunctional Materials

**DOI:** 10.3390/gels9110878

**Published:** 2023-11-06

**Authors:** Aleksandra Mikhailidi, Elena Ungureanu, Dan Belosinschi, Bogdan-Marian Tofanica, Irina Volf

**Affiliations:** 1 Higher School of Printing and Media Technologies, St. Petersburg State University of Industrial Technologies and Design, 18 Bolshaya Morskaya Street, 191186 St. Petersburg, Russia; amikhailidi@gmail.com; 2 “Ion Ionescu de la Brad” University of Life Sciences Iasi, 3 Mihail Sadoveanu Alley, 700490 Iasi, Romania; eungureanu@uaiasi.ro; 3 Innovations Institute in Ecomaterials, Ecoproducts, and Ecoenergies, University of Quebec at Trois-Rivières, 3351, Boul. des Forges, Trois-Rivières, QC G8Z 4M3, Canada; dbelosinschi@gmail.com; 4 CellON AS, Lakkegata 75C, NO-0562 Oslo, Norway; 5 “Gheorghe Asachi” Technical University of Iasi, 73 Prof. Dr. Docent D. Mangeron Boulevard, 700050 Iasi, Romania; 6 IF2000 Academic Foundation, 73 Prof. Dr. Docent D. Mangeron Boulevard, 700050 Iasi, Romania

**Keywords:** metallogels, hydrogels, cellulose, metal nanoparticles, drug delivery, tissue engineering, conductivity, wastewater treatment, biocidal properties, catalysts

## Abstract

The incorporation of the metal phase into cellulose hydrogels, resulting in the formation of metallogels, greatly expands their application potential by introducing new functionalities and improving their performance in various fields. The unique antiviral, antibacterial, antifungal, and anticancer properties of metal and metal oxide nanoparticles (Ag, Au, Cu, Cu_x_O_y_, ZnO, Al_2_O_3_, TiO_2_, etc.), coupled with the biocompatibility of cellulose, allow the development of composite hydrogels with multifunctional therapeutic potential. These materials can serve as efficient carriers for controlled drug delivery, targeting specific cells or pathogens, as well as for the design of artificial tissues or wound and burn dressings. Cellulose-based metallogels can be used in the food packaging industry to provide biodegradable and biocidal materials to extend the shelf life of the goods. Metal and bimetallic nanoparticles (Au, Cu, Ni, AuAg, and AuPt) can catalyze chemical reactions, enabling composite cellulose hydrogels to be used as efficient catalysts in organic synthesis. In addition, metal-loaded hydrogels (with ZnO, TiO_2_, Ag, and Fe_3_O_4_ nanoparticles) can exhibit enhanced adsorption capacities for pollutants, such as dyes, heavy metal ions, and pharmaceuticals, making them valuable materials for water purification and environmental remediation. Magnetic properties imparted to metallogels by iron oxides (Fe_2_O_3_ and Fe_3_O_4_) simplify the wastewater treatment process, making it more cost-effective and environmentally friendly. The conductivity of metallogels due to Ag, TiO_2_, ZnO, and Al_2_O_3_ is useful for the design of various sensors. The integration of metal nanoparticles also allows the development of responsive materials, where changes in metal properties can be exploited for stimuli-responsive applications, such as controlled release systems. Overall, the introduction of metal phases augments the functionality of cellulose hydrogels, expanding their versatility for diverse applications across a broad spectrum of industries not envisaged during the initial research stages.

## 1. Introduction

The utilization of cellulose-based products offers a variety of environmental and economic benefits. These materials, derived from wood and non-wood sources, exhibit a spectrum of characteristics that make them beneficial for diverse applications in the forms of fibers, films, hydrogels, and aerogels, as well as nanoscale materials [1,2,3,4]. The structure of cellulose hydrogels provides a superior water-holding capacity and certain properties, namely mechanical strength, porosity, and specific surface area. Additionally, an imposing quantity of reactive functional groups allows the hydrogel to capture nanoparticles and other additives. Being sustainable, biodegradable, non-toxic, biocompatible, and multi-receptive materials, bio-based cellulose materials are in high demand in such fields as medicine, pharmacology, cosmetics, food industry, environment protection, electronics and energy [5,6,7,8,9,10,11,12]. For example, in the context of disposable packaging materials, the imperative for biodegradability is evident to alleviate landfill concerns. Alternatively, when employed in biomedical applications, e.g., wound dressings, cellulose hydrogels ensure safe combustion without generating toxic by-products. Moreover, the biocompatibility of these materials is essential for medical products.

The cost-efficiency and sustainability of materials are universally paramount considerations [13]. Environmentally friendly production involves the utilization of various renewable resources as an intrinsic component of the cellulose-based polymer paradigm. Cellulose can be derived from plant-based biomass, such as wood pulp [14], fibers [15], algae biomass [16], cellulose-producing bacteria [17], agricultural [18,19] and paper waste [20].

Cellulose hydrogels are commonly prepared through the dissolution of cellulose in various solvents, including organic, inorganic, mixed, and ionic liquids, followed by gelation and solvent removal [1,5]. However, the limited solubility of cellulose restricts the choice of suitable solvents for hydrogel production. A comprehensive assessment of cellulose sources and preparation techniques, involving such solvents as N,N-dimethylacetamide (DMAc)/LiCl, dimethyl sulfoxide (DMSO)/LiCl, N-methylmorpholine-N-oxide (NNMO), and NaOH-aqueous solutions, was undertaken by the authors in the first part of the review series “*Cellulose-Based Metallogels*” [21]. The analysis revealed that DMAc/LiCl and NaOH are the more prevalent solvents, whereas two others are employed by researchers less frequently. The NaOH-aqueous system offers cost-effectiveness and environmental advantages, but demands specific pretreatment, conditions, and crosslinking. In contrast, DMAc/LiCl, while less environmentally appealing, provides a straightforward and industrially attractive hydrogel-manufacturing process.

**Figure 1:**
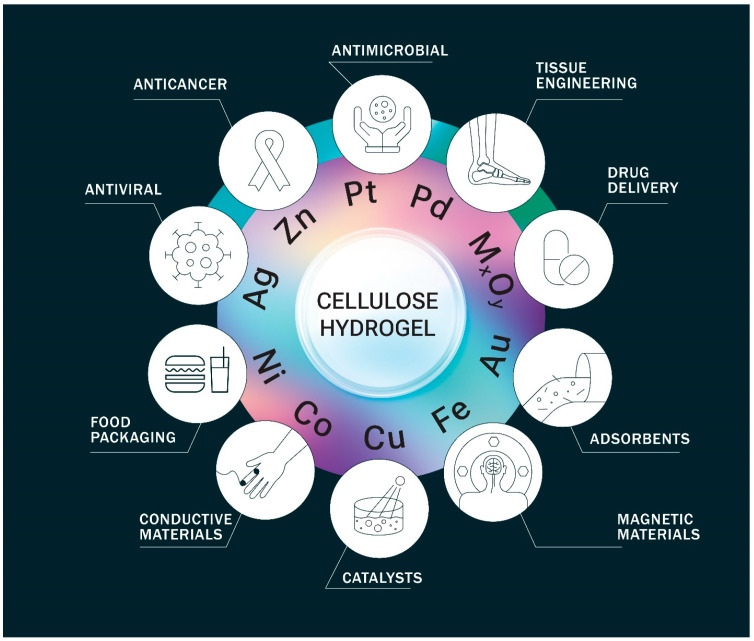
The main application fields of cellulose-based metallogels.

The incorporation of metal particles into the gel system endows the resulting metallogels with new abilities [22,23,24], summarized in Figure 1. Metal nanoparticles offer beneficial properties such as high specific surface area, diverse catalytic capabilities, unique electromagnetic and optical behavior due to their small size, and enhanced reactivity [25].

As was discussed in the second part of “*Cellulose-Based Metallogels*” [12], in which the properties of the hydrogels were compared with those of the resulting metallogels, the incorporation of metal nanoparticles into hydrogels leads to the reinforcement of mechanical strength in the resulting materials. Notably, metal-loaded hydrogels tend to exhibit improved crystallinity and thermal stability when compared to their pristine counterparts. The introduction of metal ions or nanoparticles can confer new and desirable traits, such as optical responsiveness, thermal characteristics, or biological activity, to the hydrogel matrix. The porous structure of cellulose hydrogels facilitates the exchange of metal ions, and in some cases, enables the formation of metallogels through complexation, potentially resulting in biocidal effects that endure over time [26,27].

Metallogels can be derived from ready-made cellulose hydrogels using the diffusion–reduction technique [21,24,28]. This convenient and cost-effective approach involves the use of chemical or phyto-chemical reducing agents for the nanoparticles’ formation from the precursor salt solutions. Alternatively, another chemical route to obtain cellulose metallogels relies on certain metal ions that initiate gelation and serve as cross-linking agents for cellulose [29]. While this method is appealing, it is limited to cellulose solvents that are aqueous in nature [21].

After exploring the production and characterization of cellulose metallogels in two previous parts of this review [12,21], the subsequent part will illuminate the diverse and captivating applications of these fascinating composite materials. Given the significant interest in cellulose/metal composite hydrogels, numerous review articles have been dedicated to this intriguing field of research. However, when a comprehensive review covers almost all aspects of cellulosic materials, including cellulose films, fibers, membranes, beads, and aerogels, e.g., the paper by Acharya et al. [1], the discussion of the application of the hydrogels modified with metal nanoparticles is often limited. Many hydrogel-focused reviews tend to center on a specific application, such as antibacterial properties [30], drug delivery [31], or tissue engineering [32,33], frequently delving into non-metal composites or cellulose derivatives rather than pristine cellulose-based hydrogels. In this review, we aim to fill this gap by providing a thorough examination of the metallogels derived from pristine cellulose, offering insights into their diverse applications.

## 2. Metallogel Science, Approaches, and Applications

### 2.1. Medical Application

The development of biomaterials is embracing metallogels, with such metals as Ag, Au, Zn, and others, as well as their oxides, taking the forefront. These materials are gaining traction due to their capacity to react to diverse physical and chemical cues (for instance, electrical, pH, magnetic, and light stimuli) while also offering supplementary attributes [34].

#### 2.1.1. Antimicrobial, Antifungal, and Antiviral Properties

Due to the widespread and often uncontrolled usage of antibiotics in recent decades, many microorganisms have become resistant to the effect of antibiotics, which makes it difficult to cure even the simplest infections. Metal and metal oxide nanoparticles (Ag, Cu, CuO, Cu_2_O, Au, ZnO, Al_2_O_3_, TiO_2_, Co_3_O_4_, In_2_O_3_, MgO, ZrO_2_, Cr_2_O_3_, NiO, Ni_2_O_3_, Mn_2_O_3_, and CoO) have demonstrated selective effectiveness against priority pathogens. The non-specific bacterial toxicity mechanisms of metal nanoparticles, devoid of binding to specific bacterial cell receptors, hinder resistance development and expand the range of antibacterial effects [35,36].

A number of metal and metal oxide nanoparticles (such as silver, gold, copper, titanium dioxide, and zinc oxide) have been recently introduced as antiviral agents [37,38,39,40]. As was revealed in the review by Sánchez-López et al., nanoparticles employ distinctive mechanisms of action that differ from conventional treatments. This grants them the ability to combat antibiotic-resistant bacteria and target various biomolecules, hindering the emergence of resistant strains. Concerns about potential human toxicological effects related to metal nanoparticles stem from their physico-chemical traits, dosage, and administration method, which dictate their behavior within the body [35].

Addressing this challenge, a polymer matrix can play a crucial role in managing the targeting and gradual release of nanoparticles. Hydrogels exhibit porosity, remarkable flexibility, and a capacity to retain water. These attributes create a moist, tissue-like environment, rendering it a fitting biomaterial for diverse biomedical uses [40]. These requisites are realized in cellulose hydrogels, making them an ideal carrier for metal nanoparticles. As was shown in numerous studies, introducing metals such as silver, gold, copper, and zinc, as well as metal oxides, in nanosized or colloid states into cellulose matrices confers antimicrobial and antifungal properties to the composite material [24,28,41,42,43,44,45].

An effective method for producing antimicrobial cellulose/metal composite hydrogels involves the chemical or biological reduction of the metal ions within the ready-made cellulose hydrogel [20]. For instance, hydrogels prepared from solutions of hardwood and flax powder celluloses in DMAc/LiCl demonstrated high porosity and specific surface areas. These attributes are crucial for anchoring the reduced metal nanoparticles within the cellulose matrix. The synthesis of silver and gold nanoparticles was carried out via the Turkevich method of reducing gold ions from a sodium tetrachloroaurate (III) Na[AuCl_4_] solution and silver ions from a silver nitrate AgNO_3_ solution with trisodium 2-hydroxypropane-1,2,3-tricarboxylate (trisodium citrate) as a reducing agent.

**Figure 2:**
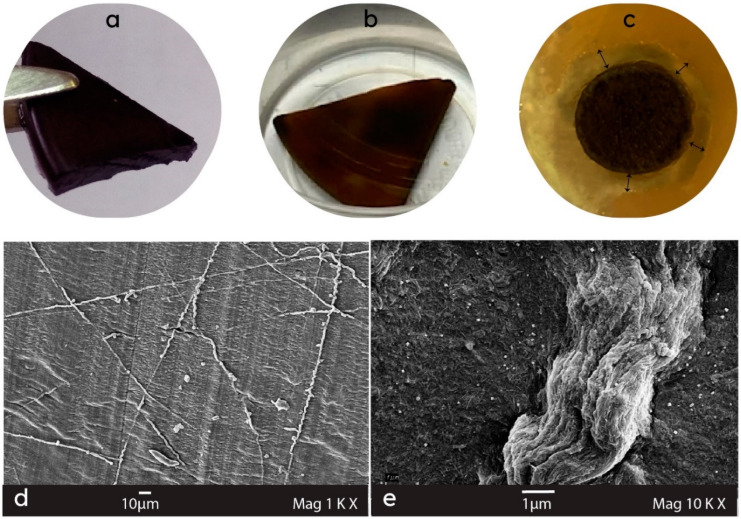
Digital photographs and SEM micrographs, respectively, of (**a**,**d**) Au/cellulose metallogel (2.9 wt.% Au), (**b**,**e**) Ag/cellulose metallogel (2.1 wt.% Ag), and (**c**) no-growth zone on agar inoculated with *E. coli* bacteria in the experiment with Ag/cellulose metallogel after 24 h of incubation.

The digital pictures of the resulting Au- and Ag-containing metallogels are presented in Figure 2 (a and b, respectively). The nanoparticles intercalated into cellulose hydrogels exhibited various shapes (spherical or rectangular) and sizes. Gold nanoparticles predominantly ranged from 40 to 120 nm, sometimes forming larger agglomerates of several micrometers (Figure 2d). A higher concentration of trisodium citrate facilitated the rapid stabilization of smaller-sized Au nanoparticles, while a lower concentration led to larger Au nanoparticles due to aggregation. Silver nanoparticle sizes ranged from 20 to 260 nm (Figure 2e), showing a more uniform size distribution for gold nanoparticles compared to silver nanoparticles [46]. It was demonstrated that cellulose hydrogels that contained from 0.30 to 5.66 wt.% of Ag or Au nanoparticles revealed antimicrobial activity against Gram-positive (*Staphylococcus aureus*) and Gram-negative (*Escherichia coli*) bacteria (Figure 2c) [24,46,47]. The biocidal activity was not notably influenced by the metal content in the given range. This suggests that there is no necessity to elevate it beyond 1 wt.%, avoiding the possible toxic effect of metal nanoparticles on humans. All examples are briefly summarized in Table 1.

Zinc and zinc oxide nanoparticles incorporated into cellulose materials have gained attention for their antibacterial and antiviral properties [48,49]. The biocidal effects of ZnO and Ag nanoparticles differ: ZnO particles are believed to react with water molecules, producing reactive oxy-radicals or hydroxyl-radicals that induce oxidative damage within bacterial cells. On the other hand, Ag nanoparticles enter bacteria or adhere to their surfaces, disrupting permeability and respiration functions [49]. The release of Zn^2+^ ions from zinc oxide nanoparticles is a primary mechanism driving their oligodynamic activity against both eukaryotic and prokaryotic microorganisms. Additionally, ZnO can generate reactive oxygen species (ROS) through electron hole formation under specific light wavelengths. These properties, coupled with the appropriate biocompatibility and biodegradability of cellulose hydrogels, have led to their increased utilization in therapeutic and cosmetic applications [50]. Hence, the bactericidal effect is not solely limited to the well-renowned yet costly silver and gold nanoparticles. Similarly, effective outcomes can be achieved with more economical metallogels incorporating affordable metals and their oxides, such as zinc and copper oxides. For example, a porous bacterial cellulose aerogel (BCA) was employed as a template for Ag, CuO, and ZnO nanoparticles. They were synthesized through the chemical reduction of the corresponding salts using sodium borohydride (NaBH_4_) as a reducing agent. Ag/BCA and CuO/BCA displayed spherical nanoparticle morphology with an average size of approximately 50 nm and 10 nm, respectively, while ZnO/BCA exhibited hexagonal morphology at around 50 nm. All metal nanocomposites demonstrated antibacterial effects against both Gram-negative *Klebsiella pneumonia* and Gram-positive *Staphylococcus aureus* bacteria [51].

Organic–inorganic metallogels synthesized by incorporating the 1,10-phenanthrocyanines zinc (II) complex into cellulose hydrogels demonstrated enduring stability, substantial water retention capacity, and porosity. The immobilization of the complex within the hydrogels facilitated interaction with the cellulose matrices, resulting in additional cellulose crystallization during composite hydrogel formation. Among electron-rich coordination compounds of d-elements (Zn(II), Pd(II), Pt(II), Co(II), and Ag(I)) grounded in 1,10-phenanthrocyanates, promising antibacterial, antifungal, and antiviral properties have been observed [52]. The metal ions’ characteristics significantly influenced the formation conditions, structure, stability, and reactivity of 1,10-phenanthrocyanine complexes of d-elements.

Another intriguing method to impart antimicrobial properties to cellulose hydrogels involves combining nanoparticles with other bactericidal agents. For instance, Gupta and colleagues introduced a cellulose/Ag metallogel infused with curcumin, a natural polyphenol known for its antimicrobial, antioxidant, and anti-inflammatory effects. The reduction of Ag nanoparticles from an AgNO_3_ solution was accomplished using an aqueous solution of curcumin/hydroxypropyl-β-cyclodextrin complex. This complex was formulated to address the hydrophobic nature of curcumin. Following reduction, the cellulose hydrogels were immersed in a suspension containing Ag nanoparticles and curcumin. The resulting material exhibited high cytocompatibility and antimicrobial activity against three common wound-infecting pathogenic microorganisms: *Staphylococcus aureus*, *Pseudomonas aeruginosa*, and *Candida auris* [53]. The synergistic effect of zinc oxide nanoparticles and propolis extracts deposited on bacterial cellulose (BC) was investigated in [54]. ZnO nanoparticles were synthesized directly on BC surfaces using ultrasound. BC/ZnO composites were subsequently infused with ethanolic propolis extracts at various concentrations. The antimicrobial synergy of BC/ZnO/propolis films was effective against *Escherichia coli*, *Bacillus subtilis*, and *Candida albicans*. However, no effect on Gram-negative bacteria or eukaryotic cells was indicated for BC/ZnO [54]. One more example is a dialdehyde cellulose–chitosan/ZnO metallogel enhanced by the synergistic effect of nanoparticles and bioactive compounds. The hydrogel matrices were embedded with ZnO nanoparticles and phyto-derived quercetin from onion peel waste. The resulting materials exhibited biocompatibility and antibacterial effects against *Staphylococcus aureus* and *Trichophyton rubrum* [55]. In another study, Anagha and colleagues confirmed the antimicrobial activity of curcumin-loaded hybrid hydrogels with ZnO nanoparticles against the same microorganisms [56].

The healing of damaged skin tissue, especially in chronic wounds, is a complex and challenging process, often aggravated by bacterial infections. Common wound dressings, designed for specific functions, may not adequately address the holistic requirements of the healing process. For instance, conventional dressings are dry and therefore cannot provide a moist environment for wound healing and do not possess antibacterial properties. Hence, there is a need for novel wound dressings that offer multifunctionality [57,58]. For this reason, cellulose hydrogels, possessing beneficial attributes suitable for wound dressing applications—such as softness, flexibility, tunable physico-chemical properties, high porosity, robust biocompatibility, and biodegradability—show promising potential in this domain [12]. Moreover, they can easily incorporate drugs and/or metal nanoparticles, bear resemblance to the natural extracellular matrix (ECM), and possess exceptional water-absorbing and water-retaining capacities [59].

The incorporation of metal phase into the cellulose hydrogels often enhances the mechanical strength, thermal stability, and swelling capacity of materials. As has been shown, nanoparticles contribute to antimicrobial and antiviral traits in metallogels, and in some instances, they encourage cell activity and growth. The porous structure of cellulose-based metallogels facilitates an extended biocidal impact [12]. For example, nanocomposite dressings (including hydrogel strips) exhibited remarkable antibacterial and antifungal effects against *P. aeruginosa*, *C. freundii*, *E. cloacae*, *E. coli*, *S. aureus*, *S. epidermidis*, *B. subtilis*, and *C. parapsilosis*. These dressings were created by incorporating silver nanoparticles into a cellulose nanocrystal (CNC) matrix. When topically applied to acute and diabetic wounds in mice, these nanocomposites exhibited improved tissue repair, achieving approximately 99% wound closure. This enhancement was attributed to reduced inflammation, increased angiogenesis, collagen deposition, and accelerated neo-epithelialization.

The synergistic action of CNCs (with high water uptake capacity) and Ag nanoparticles (acting as antimicrobial agents) led to an elevated expression of essential growth factors and collagen while simultaneously lowering levels of pro-inflammatory factors, thereby expediting the healing process. The outcomes underscore the potential of these nanoporous nanocomposites, with optimized Ag nanoparticle concentration, as effective and cytocompatible dressings for advanced wound management [60].

On the other hand, in vitro studies conducted with simulated wound fluid lack corresponding in vivo investigations, which are essential due to differences in biofilm presence, mixed bacterial species, tissue proteins, and anions [61]. There are limited supporting randomized controlled trials (RCTs) assessing various silver dressings’ impact on wound healing in contaminated and infected wounds. Although some RCTs have shown advantages like reduced wound size and faster healing rates [60,62,63], other studies have indicated no significant differences [64]. The complex in vivo wound environment, encompassing biofilms and host proteins, complicates the delivery of silver ions [61].

#### 2.1.2. Anticancer Properties

Nanoparticles of certain metals are recognized as promising anticancer agents due to their effectiveness against drug-resistant tumor cells through distinct mechanisms [65]. Au, Ag, and Zn nanoparticles [66,67,68], as well as bimetallic colloidal particles [69,70], exhibit effects both in cancer diagnosis and treatment, offering alternatives to current toxic anticancer drugs. Existing treatments produce harmful side effects and drug resistance along with rapid metabolism and clearance, limiting efficacy. Metal nanoparticles can be combined with drugs or polymer-coated for targeting cancer cells [65].

The major mechanisms by which metal nanoparticles exert their anticancer properties involve cellular uptake via endocytosis. Following this, vesicles are dispersed throughout the cytoplasm and nucleus, causing toxic effects that ultimately result in apoptosis (programmed cell death) [71,72]. For instance, silver and gold nanoparticles can induce inflammation in treated cells by activating macrophages [73]. They also possess antiangiogenic properties by blocking the completion of signaling pathways [74,75], along with antiproliferative effects attributed to the induction of genomic and cytoskeletal instability [76,77]. Size-dependent effects indicate smaller nanoparticles are more toxic, generating ROS more efficiently [78].

Gold nanoparticles can serve as delivery systems in cancer therapy [79] or are combined with therapeutic molecules [80], including genes, enhancing their efficacy against cancer cells. Their distinct photo-optical attributes enable successful utilization in both photothermal (inducing hyperthermia and eventual cell necrosis) and photodynamic (creating ROS) therapies [81,82].

Thus, metal and metal oxide nanoparticles exhibit promising potential in cancer treatment; however, their direct skin or oral application is not feasible. A carrier is essential to control the release of active nanoparticles at precise concentrations and timings. As one viable approach, nanoparticles can be incorporated within polymer hydrogels or chemically cross-linked into their networks. Cellulose’s capacity for liquid absorption and loading active substances, such as drugs and metal nanoparticles, makes it a promising material for diverse cancer therapies [83]. Hydrogels composed of hydrophilic cellulose have been extensively utilized for skin cancer treatment, providing a water-rich environment [59]. Nanocomposite hydrogels can induce controlled high temperatures via near-infrared irradiation, effectively eradicating tumor cells and inhibiting their growth [84]. Owing to their favorable mechanical, biological, and physico-chemical properties [12], they mitigate the risk of post-surgical tumor recurrence [85]. Skin cancer simultaneously couples with skin wound infection. Nanocomposite hydrogels with simultaneous photothermal antitumor and antibacterial efficacy can meet the dual demand of cutaneous melanoma treatment and skin wound healing [59]. For instance, cellulose-based metallogels with ZnO nanoparticles, enriched by quercetin and curcumin, exhibited both anticancer and antimicrobial capabilities [55,56]. Similarly, cellulose hydrogels incorporating coordination complexes of zinc, palladium, platinum, cobalt, and silver with 1,10-phenanthrocyanates demonstrated promising potential in both anticancer and antimicrobial applications [52].

#### 2.1.3. Drug Delivery

The domain of drug delivery has made remarkable strides in recent times, particularly with the rapid advancement of nanomedicine, coupled with enhanced insights into infectious and cancerous conditions [86]. One of the crucial pharmacological purposes is delivering precise amounts of drugs at pre-planned rates to provide the desired level of drugs for treatment.

Cellulose-based hydrogels that respond to various stimuli exhibit a remarkable ability to undergo rapid volume changes in reaction to environmental triggers, transitioning between collapsed and swollen states. This unique behavior renders them highly promising for biomedical applications. These environmental stimuli encompass factors like pH, temperature, redox reactions, light exposure, solvent composition, electric and magnetic fields, and even biological or biochemical signals [87]. Body temperature and variable pH ranges in different parts of the body function as external stimuli to activate the release of the drug. Cellulose hydrogels can perform as safe transport systems with desired therapeutic effects and with minimum side effects. Moreover, the results of the utilization of hydrogels in target therapy strategies obtained in clinical trials are very encouraging [31,88]. Usually, pure-cellulose hydrogels have a disadvantage of low mechanical characteristics because of the physical mechanism of formation.

Chemically cross-linked hydrogels demonstrate better properties, as a result, they are preferable. Metal and metal oxide nanoparticles can play dual roles as a complexation center to improve the cross-linking of the hydrogel, as well as an antimicrobial and anticancer component. There are a number of examples when the hydrogels for drug delivery are produced from cellulose derivatives or enhanced with a different component, resulting in a hybrid hydrogel. For instance, a Cu-based metal/organic framework was produced for the encapsulation of ibuprofen to reduce the side effect of this drug on the gastrointestinal tract. In this material, a carboxymethyl cellulose hydrogel bead demonstrated better protection of ibuprofen against stomach acid and a high stability of drug release for a long period of time [89].

In another study, leptin, a hormone that helps to maintain normal weight, was entrapped within methyl cellulose hydrogels, with the incorporation of gold nanoparticles. The light-triggered degradation of hydrogels was used to improve accuracy and efficiency for sustained and controllable release. The incorporation of gold nanoparticles into methyl cellulose hydrogels led to a tunable light irradiation response. The study revealed that thermosensitive hydrogels are suitable for loading multimodality therapeutic agents to enhance the bioactivity of leptin for obesity therapy [90].

One more complex example involved another natural polymer—chitosan—is described in [91]. A nanohybrid hydrogel of L-histidine conjugated chitosan, phyto-synthesized zinc oxide nanoparticles, and dialdehyde cellulose was successfully used as a drug delivery carrier for the polyphenolic drugs naringenin, quercetin, and curcumin. The hydrogel demonstrated a peak loading efficiency at 90.55%, 92.84%, and 89.89%, respectively. The maximum drug release was favorably observed at optimal drug loading and at pH 5. Histidine–chitosan conjugation stabilized the hydrogel and enabled sustained drug delivery.

Prominent antimicrobial activity against Staphylococcus aureus and Trichophyton rubrum strains was anticipated to develop through a synergistic formulation. Significant biocompatibility with L929 cells demonstrated support for normal cell survival. Anticancer analysis of A431 cells displayed excellent cytotoxicity, with a 15- to 30-fold increase using a hybrid carrier, in contrast to free polyphenol drugs [91].

On the other hand, there are examples of a cellulose hydrogel being applied without any enhancements other than metal nanoparticles. Thus, regenerated cellulose obtained from sugarcane bagasse was used for hydrogel preparation with zinc oxide nanoparticles photosynthesized from musk melon seed extract. For a drug delivery study, curcumin was selected as the model drug for its appealing anticancer and antimicrobial activity. The drug release was performed under varying pH and initial drug loading concentrations. Polymer swelling as the drug release mechanism was the best option according to the results [56].

Incorporating metal nanoparticles into hydrogels is not the only approach to design drug delivery metallogels; metal–organic compounds can also be integrated into hydrogels for these applications. Belonging to the class of metal–organic frameworks, zeolitic imidazolate frameworks (ZIFs) are characterized by the coordination of metal clusters with organic ligands to create three-dimensional structures. Usually, ZIFs are composed of tetrahedrally coordinated transition metal ions (e.g., Fe, Co, Cu, and Zn) connected by imidazolate linkers. Addressing the challenge of loading hydrophobic drugs into hydrophilic nanocellulose-based hydrogels, a metal–organic approach was employed. Polydopamine was auto-polymerized on cellulose nanofibril (CNF) hydrogel surfaces, forming a PCNF composite hydrogel. Subsequently, zeolitic imidazolate frameworks-8 (ZIF-8) nanoparticles were grown within the PCNF composite hydrogel structure. The resulting material exhibited robust mechanical strength, and efficient drug delivery characteristics were achieved. The ZIF-8/PCNF composite hydrogel demonstrated prolonged drug release times and anticancer effects, highlighting its potential as a promising biomaterial for drug delivery applications [92].

#### 2.1.4. Tissue Engineering

Researchers in the field of tissue engineering and regenerative medicine are addressing the issues of organ scarcity and biocompatibility by exploring scaffolds as a substitute for transplants [93]. Tissue scaffolds play an important role in regeneration by mimicking the ECM in natural tissues and providing a suitable 3D space for cell proliferation and differentiation. Therefore, scaffold materials must have reasonable biocompatibility, mechanical properties, swelling behavior, and porosity [94].

Cellulose-based hydrogels have found extensive applications in tissue and biomedical engineering, leveraging their impressive properties, such as remarkable swelling capabilities, strong water absorbency, mechanical resilience, and compatibility with biological tissues, all of which facilitate effective binding. These hydrogels contain a large number of hydroxyl groups, which are conducive to the formation of composite hydrogels with other polymers, metals (usually Au and Ag), or small molecules. They enable the regeneration of various tissues, such as bone, cartilage, heart, blood vessel, nerve, and liver, among others [9,94].

In the context of skin regeneration, cellulose hydrogels offer an added advantage: they possess the ability to promote hydration healing, exhibit suitable oxygen permeability, absorb wound exudates, enhance epithelialization, and create an environment conducive to tissue regeneration. An example is the BC/acrylic acid hydrogel obtained through electron beam irradiation. This material exhibited a porous network structure, remarkable swelling capacity (4000–6000%), and high water vapor transmission rate (2175–2280 g/m^2^ per day). The hydrogel displayed biocompatibility in L929 cell viability tests. In vivo trials on rats demonstrated that this hydrogel expedited wound healing, facilitated epithelialization, and accelerated fibroblast proliferation in comparison to the control group. These outcomes highlight the potential of BC/acrylic acid hydrogels as promising materials for burn dressings [95].

The integration of metals, namely silver and gold, typically enhances the physical and chemical characteristics of cellulose hydrogels [12,93]. Metal nanoparticles exhibit inherent bioactivity and, as previously described, natural antibacterial, antiviral, and anti-inflammatory capabilities. In the study conducted by Zulkifli et al., an antimicrobial hydroxyethyl cellulose/Ag nanoparticle scaffold promoted the growth and proliferation of human fibroblasts. It was suggested that the surface roughness of the scaffold due to the presence of Ag nanoparticles contributed to enhanced cell adhesion and proliferation [96].

**Table 1:** The biological properties of cellulose-based metallogels.

Nanoparticle	Hydrogel Matrice/Additives	Biological Effect	References
Ag	Flax, cotton, and hardwood powder cellulose	Antibacterial activity against *S. aureus* and *E. coli*.	[24,46,47]
	Bacterial cellulose/PVA (2:5 wt.)	Antibacterial activity against *E. coli* and *S. aureus*. Promotion of the growth of new blood vessels. Anti-inflammatory activity. Accelerating wound healing.	[41]
	Microcrystalline cellulose	Antibacterial activity against *E. coli* and *S. aureus*.	[45]
	Bacterial cellulose	Antibacterial activity against *K. pneumonia* and *S. aureus*.	[51]
	Bacterial cellulose/aqueous curcumin	Cytocompatibility, wound-healing properties, and antimicrobial activity against *S.aureus, P.aeruginosa*, and *C. auris*.	[53]
	Hydroxypropyl-β-cyclodextrin complex	Tissue repair and wound healing via a decrease in inflammation; increase in angiogenesis, collagen deposition, and rate of neo-epithelialization; and cytocompatibility. Antimicrobial activity against *P. aeruginosa*, *C. freundii*, *E. cloacae*, *E. coli*, *S. aureus*, *S. epidermidis*, *B. subtilis*, and *C. parapsilosis*.	[60]
	Cellulose nanocrystals isolated from *Syzygium cumini* leaves	Low toxicity to hFB cells.	[96]
	Hydroxyethyl cellulose	Antibacterial activity against *E. coli* and *L. monocytogenes*. The composite did not significantly reduce the viability of Caco-2 and FHC colon cells.	[97]
Cu	Cellulose nanofibrils	Effective ibuprofen drug carrier with controlled release in the gastrointestinal tract. Low toxicity against Caco-2 cells.	[89]
CuO, Cu_2_O, and Cu	Carboxymethyl cellulose/ibuprofen	Antibacterial activity against *E. coli* and *S. aureus*. Biocompatibility with HaCaT cells.	[42]
CuO	Carboxymethyl cellulose, hydroxypropylmethyl cellulose	Antibacterial activity against *K. pneumonia* and *S. aureus*.	[51]
Au	Bacterial cellulose	Stimuli-responsive thermosensitive carriers for therapeutic agents to enhance the bioactivity of leptin for obesity therapy.	[90]
	Methylcellulose/leptin	Antibacterial activity against *E. coli* and *P. aeruginosa*; biocompatibility. Promotion of wound repair.	[43]
	Bacterial cellulose/4,6-diamino-2-pyrimidinethiol	Antibacterial activity against *S. aureus* and *E. coli*.	[46]
ZnO	Flax and hardwood powder cellulose	Antibacterial activity against *E. coli* and *S. aureus*.	[49]
	Bacterial cellulose	Antifungal activity towards phytopathogen *F. oxysporum*. Reduced the wilt disease symptom incidence of pepper plant.	[44]
	Cellulose from watermelon peel waste	Antibacterial activity against *K. pneumonia* and *S. aureus*.	[51]
	Bacterial cellulose	The synergetic antimicrobial effect against *E. coli*, *B. subtilis*, and *C. albicans*. Without propolis extract, BC/ZnO hydrogels had no influence on Gram-negative and eukaryotic cells.	[54]
	Bacterial cellulose/ethanolic propolis extracts	Effective quercetin drug carrier. Antimicrobial activity against *S. aureus* and *T. rubrum*. Biocompatibility and anticancer properties against normal L929 murine fibroblast cells and A431 human skin carcinoma cell lines, respectively.	[55]
	Chitosan/dialdehyde cellulose derived from Sugarcane bagasse/phyto-derived quercetin	Effective curcumin drug carrier. Antimicrobial activity against *S. aureus* and *T. rubrum*.	[56]
	Sugarcane bagasse cellulose/curcumin	Stimuli-responsive (pH) carriers for therapeutic agents. Antimicrobial activity against *S. aureus* and *T. rubrum*. Biocompatibility towards L929 cells. Anticancer activity towards A431 cells.	[91]
	Chitosan/dialdehyde cellulose derived from Sugarcane bagasse/L-Histidine/Naringenin, quercetin, and curcumin	Antibacterial activity against *E. coli*, *Salmonella*, *L. monocytogenes*, and *S. aureus*. A decline in the capacity and virulence of microorganisms to pose infections.	[98]

### 2.2. Food Packaging

Packaging materials serve multiple purposes, with the primary function being to safeguard products against diverse environmental factors: mechanical stress, gases and vapors, moisture, light, temperature, microbes, and contamination. The selection of materials considers their potential to provide protection, as well as facilitate transportation, enhance presentation, and convey consumer information. Packaging may also encompass functional components for extending the shelf-life (active packaging) [99].

Conventional plastic food packaging is primarily composed of synthetic polymers and is notoriously difficult to recycle. Challenges like food contamination, the presence of paper stickers, and the use of composite plastics, e.g., C/PP (composite polypropylene), further complicate the recycling process. Moreover, synthetic plastic packaging takes an extensive amount of time to decompose in landfills, eventually breaking down into harmful microplastics. To address these issues, the transition from petroleum-based plastics to biodegradable cellulose films and hydrogels appears to be a promising direction for advancing the packaging industry.

Being effective adsorbent materials, non-toxic, and biodegradable [11,12], cellulose hydrogels can be applied in food packaging to control the humidity and water activity of food, provide antibacterial protection, as well as act as a food quality indicator due to the ability to change color at different pH levels. Such broad possibilities are achieved mainly by filling the porous network of cellulose hydrogels with various additives. The antimicrobial properties of hydrogels are gained by loading natural cytotoxic substances (such as curcumin, quercetin, and grapefruit seed extract) or embedding metal nanoparticles (Ag and ZnO), and can also be achieved by combining these types of antimicrobial agents (Table 1) [11]. For example, biodegradable BC films modified with ZnO nanoparticles and propolis extract with antimicrobial properties were designed for food packaging [51]. Not only antimicrobial, but also antioxidant, UV-blocking, oxygen-scavenging, and water vapor permeability effects, as well as a low environmental impact, are among the benefits of cellulose-based composite materials [100].

Since packaging usually comes into contact with edible products, metal nanoparticles can pass into food. Therefore, usually, it is important for the content of nanoparticles in hydrogels to be very low. As noted earlier, about 1 wt.% of silver or gold nanoparticles is sufficient for the metallogel to have antimicrobial activity [24,46]. One of the recent studies on the cytotoxicity of silver ions released from cellulose carriers confirmed that CNF/Ag composites with an average Ag nanoparticle size of 10 nm did not significantly reduce the viability of Caco-2 and FHC colon cells, although the uptake of Ag nanoparticles through an endosomal mechanism was observed [97]. Also, it was proved that Ag and TiO_2_ nanoparticles releasing metal ions from packaging into its contents was insufficient to cause harm to human cells [100]. Moreover, another study suggests sulfated CNF/ZnO bio-nanocomposites are a novel preservative to inhibit microbial growth and repress the synthesis of exotoxins in the food industry [98]. These findings indicate that although there are relatively fewer applications of hydrogels in food packaging compared to films, cellulose/metal composites hold potential as antimicrobial materials for use in active food packaging systems.

### 2.3. Wastewater Treatment

Nowadays, a considerable number of water bodies around the globe are significantly polluted and are assessed as environmentally unfavorable. There are complex challenges posed by sites leaking hazardous waste, contaminated sediments, and the atmospheric deposition of acidifying and toxic substances, in addition to agricultural pollution sources, which also contribute to transboundary pollution. More than 80% of sewage generated by human activities is discharged into rivers and oceans without any treatment, which results in environmental pollution and more than 50 diseases [101]. A large proportion of the world’s population has to use water that does not meet hygienic requirements; moreover, contaminants pose a vital threat to aquatic ecosystems. The biggest contribution to water pollution is made by such contaminants as heavy metals, nitrogen and organic substances, fertilizers, pesticides, sediment, dyes, and oil, which increase the chemical oxygen demand of water bodies [102,103,104].

Activated carbon is widely utilized in commercial purification systems for wastewater treatment due to its exceptional adsorption capacity. Nevertheless, various unconventional, cost-effective, and renewable cellulose-based adsorbents are being suggested as alternatives for contaminant removal [105]. Cellulose gels offer beneficial properties, namely a unique structure, an elevated specific surface area, porosity, and a high density of functional groups, making them well suited for the effective removal of various pollutants from wastewater as well as for the recovery of precious or hazardous metals from wastewater in mining, electroplating, and metal processing [104,106]. The integration of inorganic compounds, e.g., metal and metal oxide nanoparticles, into cellulose gels enhances their contaminant removal capacity and photocatalytic performance while also imparting specific affinities toward certain pollutants [107]. This approach can effectively target the range of aquatic pollutants listed above, including organic dyes [108], pharmaceuticals [109], and specific anions [48]. To enhance sorption capabilities, hydrogels are sometimes transformed into aerogels [110] or modified through polymer grafting [111,112].

**Figure 3:**
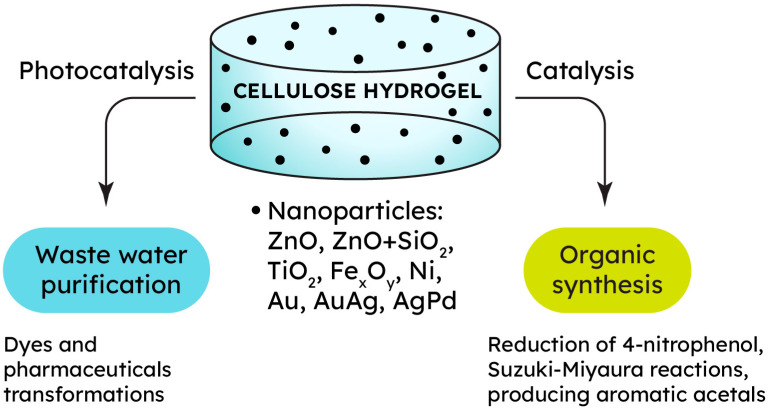
Catalytic properties of cellulose-based metallogels for different applications.

Metallogels are characterized by two main methods of water purification. The first method involves chemically transforming a dangerous organic pollutant into a simpler, less toxic substance. In addition to reducing the hazard class of wastewater, the advantage is to reduce the level of chemical oxygen demand in such wastewater. The metal phase in a cellulose metallogel can provide this material with catalytic activity in the reactions of such “chemical” water purification (Figure 3). For example, zinc oxide reveals outstanding photocatalytic performance, making it suitable for degrading complex organic compounds such as dyes and pharmaceuticals [113]. Thus, a ZnO/cellulose composite was synthesized by regenerating a cellulose hydrogel from a NaOH/urea/H_2_O (7:12:81) solution, followed by immersion in an ethanol solution of zinc acetate. The composite Zn^2+^-loaded hydrogel was then freeze-dried, and the resulting aerogel was calcined to produce flower-like ZnO structures inside the cellulose matrix. Rhodamine B (Rh B) dye served as a model compound to evaluate the photocatalytic degradation rate in this study. Upon exposure to UV light, a photocatalytic reaction was triggered, leading to a significant decrease in Rh B concentration. After 180 min, the degradation rate of Rh B using the ZnO/cellulose composite and ZnO without the hydrogel reached 95.2% and 45.2%, respectively. This suggested that the cellulose hydrogel acted as a microreactor, enhancing the catalyst’s activity twofold [113].

Ren and colleagues reported a photocatalytic composite cellulose-based hydrogel with ZnO and SiO_2_ particles that enhanced the degradation efficiency of methylene blue dye under light irradiation [108]. In this system, SiO_2_ not only acted as a cross-linking agent to enhance the mechanical strength and stability of the hydrogel but also promoted the photocatalytic properties of ZnO via transferring the electron hole pairs due to its surface state. The degradation efficiency of the methylene blue dye under light irradiation by the cellulose-based composite hydrogel was 95% in 120 min and the removal ratio remained as high as 90% after eight degradation cycles [108].

Another example is a cellulose hydrogel with TiO_2_ for efficiently and safely processing wastewater. The composite TiO_2_/cellulose microsphere produced via cellulose mineralization revealed an adsorption capacity of 91.7 mg/g on methylene blue, being reusable due to its pH-sensitiveness. More interestingly, the composite exhibited an auto-accelerating process in decomposing Rh B where the degradation intermediate could act as the receptor of photo-regenerated holes to improve the photocatalytic activity. Furthermore, the TiO_2_/cellulose microsphere intermediate adsorbent exhibited visible light responsive characteristics, achieving a removal ratio of 86.3% for tetracycline [109].

In addition to the photocatalytic decomposing of dyes and pharmaceuticals, cellulose-based metallogels can also adsorb molecules or particles of contaminants, such as heavy metal ions and dyes. This second method provides a fundamentally different but equally effective mechanism for wastewater purification. For example, a superabsorbent composite hydrogel for dye removal was suggested by Hosseinzadeh and Javadi. Magnetic iron oxide nanoparticles were synthesized in situ and integrated into a polymer hydrogel composed of carboxymethyl cellulose and poly(acrylic acid). Batch adsorption of crystal violet dye onto the hydrogel was demonstrated in various conditions: pH, dye concentration, and temperature [114]. A magnetite-containing composite hydrogel was investigated for the removal of Pb (II) ions from aqueous solutions. The magnetic hydrogel beads were composed of carboxylated cellulose nanofibrils, amine-functionalized magnetite nanoparticles (Fe_3_O_4_), and a blend of poly(vinyl alcohol) and chitosan, prepared using an instantaneous gelation method. The experimental findings indicated that the composite hydrogels displayed a high adsorption capacity for Pb^2+^ ions, reaching 171.0 mg/g. The presence of carboxylate groups on the cellulose nanofibrils’ surface played a crucial role in the adsorption process. Moreover, the hydrogels demonstrated easy regeneration in a weak acid solution, maintaining an adsorption effectiveness of 90% after four cycles [115]. In these examples, the primary absorption mechanism was driven by electrostatic interactions, encompassing both attractive and repulsive forces between charged entities (metal and polymer functional groups), including interactions between ions of opposite charges (cation–anion) as well as those with similar charges (cation–cation or anion–anion) [116].

The absorption properties of cellulose hydrogels can be improved not only through the incorporation of metal nanoparticles but also by introducing different metal compounds. A notable example involved the development of cellulose/titanate nanotube hydrogel microspheres for treating oily heavy metal wastewater. The gel-functionalized titanate microspheres were synthesized via a sol–gel process using cellulose dissolved in an ionic liquid. These self-cleaning microspheres demonstrated the capacity to adsorb up to 176.4 mg/g of Cu (II) ions through electrostatic interaction and the ion exchange mechanism. This approach holds the potential to be extended to other adsorbents, offering a viable solution for addressing wastewater treatment across different sources [117]. In a different instance, an Ag/AgCl-incorporated cellulose hydrogel demonstrated exceptional capability in efficiently degrading methyl orange when exposed to visible light, thanks to the inherent photocatalytic property of the encapsulated particles. The configuration of the hydrogel network played a pivotal role in shaping the morphology of the Ag/AgCl particles. Repeated cycle experiments highlighted the hydrogel’s commendable photocatalytic stability during multiple uses [118].

Thus, cellulose-based metallogels serve as dye-degrading catalyst systems with exceptional dispersion and active site exposure as well as heavy metal absorbents, showcasing impressive capabilities for effective and secure water treatment. The presence of charged functional groups enhances the electrostatic-driven absorption mechanism; that is why cellulose derivatives are frequently used as a basic or supplementary component of hydrogels. While cellulose-based materials are established adsorbents, their potential is restricted by such factors as production costs, environmental impacts, modification methods, and regeneration/reusability challenges [106]. Future research and development should focus on addressing these issues to advance the utilization of cellulose-based adsorbents and their surface functionalization.

### 2.4. Catalysis

The catalytic potential of cellulose metallogels extends beyond wastewater treatment. These materials hold promise in various catalytic processes, showcasing their versatility in promoting chemical transformations (Figure 3). The unique combination of cellulose’s structural attributes and the catalytic properties of metals creates a platform for tailored and efficient catalysis. Due to the large specific surface area, metal nanoparticles demonstrate fascinating catalytic reactivity in numerous reactions of organic synthesis. Additionally, hydrogel network channels are beneficial to the mass transfer process of liquid phase catalysis, creating relatively stable sites for the catalytic process [119]. When noble metals are exploited for catalytic purposes, the question of recovery and recyclability becomes crucial due to the low earth abundance and high cost of noble metals.

Lin applied a NaOH/urea regenerated cellulose hydrogel as a matrix for nanoparticles of gold; the method allows controlling the size of the particles by varying the concentration and the reaction temperature [120]. During the process of synthesizing nanoparticles within the hydrogel through the chemical reduction method, it was observed that higher concentrations of [AuCl_4_]^−^ resulted in a significant increase in the average diameter of the formed Au nanoparticles. Additionally, as the reaction temperature was elevated, there was a noticeable decrease in the average diameter of the Au (0) nanoparticles. This underscores the influence of the concentration of a precursor salt and reaction temperature on the size characteristics of the produced nanoparticles within the hydrogel matrix. The Au/cellulose metallogels exhibit potential utility as effective heterogeneous catalysts in the reduction of 4-nitrophenol by NaBH_4_. Notably, the catalytic activity is enhanced with smaller-sized Au nanoparticles. Furthermore, these Au/cellulose hydrogels can be conveniently isolated following the catalytic reaction [120]. Another cellulose-based hydrogel incorporated with bimetallic nanoparticles (AuAg, AuPd, and AgPd) was introduced by Liu as a catalyst for the same reaction of 4-nitrophenol reduction and Suzuki–Miyaura coupling reactions. The bimetallic nanocomposite hydrogels demonstrated the ability to be recycled for more than 10 cycles while maintaining their effectiveness [121]. In another study, bacterial cellulose aerogels were selected as the substrate for supporting and dispersing metal nanoparticles, namely Cu and Ni. It was found out that adsorption driven by swelling could effectively govern both the size and distribution of the metal nanoparticles. Cu and Ni nanoparticles were successfully embedded within the bacterial cellulose network, with Cu particles being smaller than Ni particles. The metal-loaded catalysts demonstrated favorable catalytic performance in the reduction of 4-nitrophenol. Notably, the optimal sample prepared with a 0.5 wt.% CuSO_4_ solution exhibited rapid completion of the reduction process within 8 min. Moreover, this catalyst exhibited remarkable stability and reusability [122].

Nanoporous cellulose nano-sponges were synthesized by combining TEMPO-oxidized cellulose nanofibers with branched polyethyleneimine and citric acid in a water solution. These materials were then loaded with Cu (II) or Zn (II) metal ions through the metal chelation properties of the cellulose. The resulting composites exhibited exceptional performance as heterogeneous catalysts in facilitating reactions between aromatic aldehydes and alcohols, producing aromatic acetals. Under optimized conditions, these catalysts achieved conversion rates exceeding 90%, with nearly complete selectivity towards acetal products, minimizing or eliminating the formation of carboxylic acid by-products. Furthermore, these metal-loaded cellulose nano-sponges could be recycled up to five times without losing their catalytic activity [123]. A composite hydrogel derived from wheat straw cellulose and feather protein using [Bmim]Cl ionic liquids, which contained copper nanoparticles, was proposed as a catalyst for the reduction of 2-nitrobenzoic acid to 2-aminobenzoic acid. Impressively, the catalytic activity of this composite material remained at 99.02% over five cycles of utilization and 90.60% after 30 days of storage, highlighting its exceptional recyclability and stability [119].

As research in this field continues to advance, it is becoming evident that composite cellulose/metal materials have the capacity to develop catalytic processes, offering environmentally friendly and economically viable solutions for catalytic applications in organic synthesis.

### 2.5. Conductive Materials

Conductive hydrogels based on natural biopolymers hold immense promise in the realm of wearable and stretchable sensing devices because they combine the reliable long-term healing capabilities of sensors with environmental degradability/recyclability for reducing electronic waste [124,125,126]. These hydrogels leverage the renewable and non-toxic nature of biopolymers, along with their biocompatibility, while also harnessing the exceptional flexibility and conductivity.

Unlike conventional flexible substrates originating from petroleum-derived polymers, conductive hydrogels obtained using natural polymers, including cellulose, possess a unique advantage. Their continuous cross-linked polymer networks contribute to mechanical flexibility, while the abundant water content facilitates uninterrupted ionic transport, resulting in an exceptional combination of stretchability and conductivity [125]. Conductive hydrogels, derived from cellulose, find practical applications as flexible strain sensors, particularly in the realm of wearable devices aimed at monitoring human movement patterns [126]. For instance, a research group led by Fu developed a multifunctional strain sensor for healthcare-monitoring systems by engineering a TEMPO-oxidized nanofibrillated cellulose pre-reinforced gelatin nanocomposite hydrogel infused with Fe (III) ions. Employing a multi-dynamic interaction strategy, they achieved the synchronized modulation of both bulk and interfacial interactions, resulting in impressive properties including high compressive stress (1310 kPa), self-healing capabilities, and electrical conductivity. Leveraging these attributes, a gelatine/NFC/Fe^3+^ hydrogel was harnessed as a multifunctional strain sensor with a gauge factor as high as 2.24 under 6% strain and a compressive sensitivity of 1.14 kPa^−1^ under 15 kPa. This sensor exhibited potential applications in manufacturing electronic skin to accurately detect subtle body movements, handwriting, and personal signatures. Notably, the sensor also displayed reliable self-healing properties for long-term usage, environmental degradability, and complete recyclability to reduce electronic waste [124]. Several flexible strain sensors with Ag nanoparticles in the matrices of cellulose hydrogels, both antibacterial and conductive, were recently suggested [127,128]. One of the devices was designed from silver nanoparticles prepared via the solid-state reduction of hydroxyethyl cellulose and compounded into a chemically cross-linked hydrogel with polyacrylamide. The sensor revealed mechanical properties of 0.12 MPa at 704.33% strain, with the highest gauge factor reaching 4.73 in the range of 125–200% strain [127]. In the second device, the in situ generation of silver nanoparticles on the cellulose skeleton was easily achieved via a heating process. This process not only offered excellent antibacterial properties to the hydrogels but also improved the mechanical properties of the hydrogels due to the elimination of the negative effect of silver nanoparticle aggregation. The tensile strength and toughness were able to reach as high as 2.0 MPa and 11.95 MJ/m^3^, respectively. Moreover, a high detection range (up to 1300%) and sensitivity (gauge factor = 4.4) were observed in the strain sensors [128].

A fundamentally different approach to the production of sensors using cellulose hydrogels and metals is described in the following example. An inorganic nanotube aerogel that has a framework consisting of hollow nanotubes was reported as a new class of porous material by Korhonen and co-authors. Firstly, nanocellulose hydrogels were prepared; then, they were freeze-dried to obtain a highly porous percolating network of cellulose aerogel. Titanium dioxide, zinc oxide, and aluminum oxide were applied on the aerogel templates using the atomic layer deposition (ALD) technique. Uniform oxide layers were readily deposited via ALD onto the fibrils, leading to organic–inorganic core-shell nanofibers. Once the composite was prepared, it was calcined at 450 °C to remove the organic core, leading to purely inorganic self-supporting aerogels consisting of hollow nanotubular networks. A titanium dioxide nanotube network was successfully applied as a resistive humidity sensor with a fast response [129].

The diverse applications of cellulose-based hydrogels integrated with metals and metal oxides illustrate their potential in producing multifunctional strain sensors with superior performance, environmental sustainability, and promising prospects for wearable technologies.

### 2.6. Magnetic Materials

Magnetically responsive cellulose materials represent a class of smart materials characterized by the incorporation of magnetic nanoparticles into a polymer matrix. This combination enables these materials to dynamically alter their physical properties in response to external magnetic fields.

**Figure 4:**
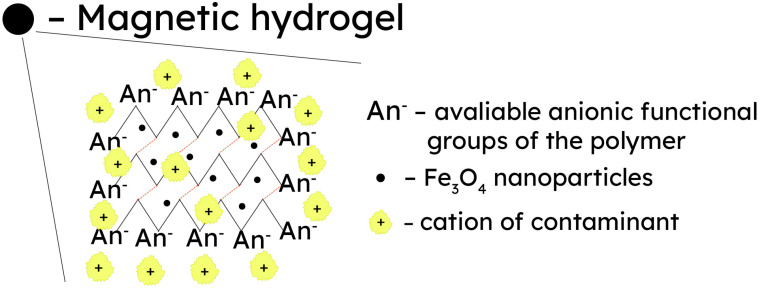
An overview of treating heavy metal-polluted wastewater by harnessing the magnetic responsiveness of metallogels.

Cellulose hydrogels can be made responsive to magnetic stimuli for applications in the areas of biotechnology/biomedicine, healthcare, environmental protection, catalysis, and magnetic resonance imaging [130,131]. Different iron oxides, namely magnetite (Fe_3_O_4_) and hematite/maghemite (Fe_2_O_3_), in the form of nanoparticles are commonly employed in the fabrication of magnetic cellulose materials. This is achieved by immersing a pre-formed hydrogel into a precursor salt solution, which is then subjected to a pH shift or a chemical reaction to initiate particle formation [132], resulting in the structure presented in Figure 4. For example, the one-step co-precipitation method was applied to produce magnetic cellulose hydrogels by dissolving cellulose in NaOH/thiourea/urea and combining the cellulose solution with pre-formed magnetic particles or via the dropwise addition of a solution of FeCl_3_ and FeCl_2_ into the cellulose solution. The NaOH in the cellulose solution acted as the precipitant of iron oxide nanoparticles, and cellulose was used as the template to promote the growth of nanoparticles. The Fe_2_O_3_ nanoparticles were dispersed in the polymer due to the synergistic effect. Magnetometric measurements revealed that the resultant cellulose/Fe_2_O_3_ composites exhibited sensitive magnetic-induced behavior and could be easily separated from an aqueous solution through the external magnetic field [133]. Another instance involved the preparation of a magnetic metallogel with hydroxypropyl cellulose and maghemite nanoparticles (~7 nm in size) at pH 13 without a chemical cross-linker. The metallogel, obtained due to reversible physical gelation and therefore sensitive to variations in pH, exhibited a magnetic moment when subjected to an external magnetic field and displayed superparamagnetic behavior. Significantly, hydroxypropyl cellulose can accommodate up to 100% of its weight in iron (II) oxide, leading to the formation of a complex structure that imparts a substantial magnetic moment to the gels [130,134]. Lin and collaborators reported a one-pot method for the synthesis of magnetic β-cyclodextrin/cellulose hydrogel beads, which exhibited rapid swelling–deswelling properties under an external magnetic field due to the incorporation of Fe_3_O_4_ nanoparticles. Cytotoxicity tests confirmed the excellent biocompatibility of the developed hydrogel [135]. In another study, the same research group suggested cellulose and chitosan coatings for Fe_3_O_4_ nanoparticles for the removal of heavy metal ions from solutions [136].

Not only iron oxide nanoparticles, but also ferrites, have been reported to grant magnetic properties to cellulose hydrogels. For example, a manganese ferrite MnFe_2_O_4_/cellulose magnetic composite aerogel was manufactured through the incorporation of MnFe_2_O_4_ into a regenerated cellulose hydrogel matrix, followed by CO_2_ supercritical drying. The hybrid aerogel displayed superparamagnetic behavior, with a maximum saturation magnetization of 18.53 emu/g. Moreover, the aerogel exhibited impressive specific surface areas ranging from 236 to 288 m^2^/g and a total pore volume of 0.55 to 0.88 cm^3^/g [137]. Indeed, aerogels, known for their highly porous nature, high internal surface, and low density frequently, offer a plethora of advantageous attributes [2,130]. Cobalt ferrite (CoFe_2_O_4_) was also suggested as a magnetic filler for cellulose aerogels [138,139,140]. Nevertheless, for potential biomedical applications of magnetic composites, iron oxides are a more favorable choice due to their suitability for in vivo use, unlike cobalt and nickel nanoparticles, which can exhibit toxicity concerns [132].

Cellulose-based hydrogels with magnetic properties due to iron oxide particles are predominantly applied for the removal of heavy metals from wastewater by using the carboxylate groups of cellulose as an active site for metal ion retention. In these composites, magnetic properties enhance the adsorption capacity and recovery performance of cellulose through the use of the external magnetic field [141].

**Figure 5:**
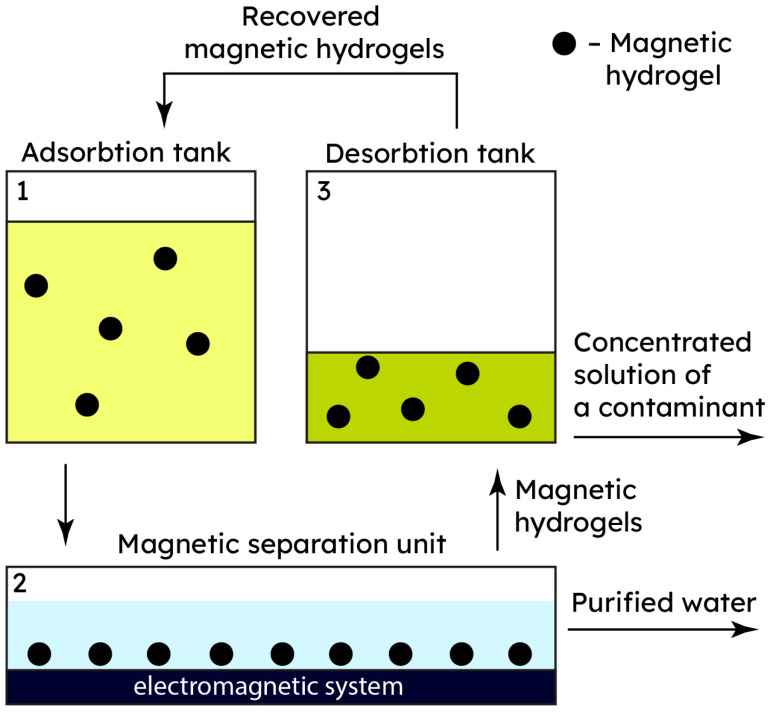
The flowsheet for treating heavy metal-polluted wastewater using the magnetic responsiveness of metallogels.

The wastewater treatment process involves several stages, as depicted in Figure 5. Initially, wastewater and a magnetic hydrogel are introduced into an adsorption tank, allowing adsorption to occur under stirring. Subsequently, the mixture proceeds to an electromagnetic device for the separation of the magnetic hydrogel using its superparamagnetic property. This separation process is swift, taking around 5 min. The purified wastewater exits the separation unit, while the magnetic hydrogel is recovered in the desorption tank. Comparative economic analysis indicates that the pilot system employing a magnetic hydrogel outperforms conventional wastewater treatment methods, namely chemical reduction and precipitation, in terms of cost-effectiveness (up to 4 times less expensive) and ecological impact (no chemical sludge is generated during the sorption and desorption processes).

The adsorption mechanisms were identified as involving electrostatic attraction and hydrogen bonding for dye adsorption, while for the adsorption process of heavy metal ions, a dominant role was played by the anionic groups of the hydrogel [142]. The presence of cellulose ethers or chitosan can further enhance the number of anionic groups, thereby augmenting the adsorption capacity towards heavy metal ions [134,141,142,143,144,145].

The mechanical properties of cellulose-based metallogels can be improved with special additives, such as CNC or quaternized xylan [146], as well as with the inorganic phase of iron oxides or ferrites [140].

The practical aspects of the use of magnetic hydrogels for wastewater treatment is comprehensively discussed in [147]. Although the composite magnetic hydrogel applied in that study was not cellulose-based, it contained Fe_3_O_4_ nanoparticles embedded within the polymer matrix during the synthesis of the hydrogel. The study provided crucial insights into a pilot wastewater purification technique that could potentially be adapted for cellulose-based magnetic metallogels.

Furthermore, the resulting concentrated heavy metal solution after desorption could be reintegrated into production processes without further purification. Hence, magnetic purification with the help of polymer composite hydrogels with magnetic nanoparticles presents a promising approach applicable for pilot-scale testing and future upscaling in the industry.

## 3. Conclusions

In conclusion, cellulose-based metallogels have emerged as a versatile and promising class of materials with a wide array of applications in different fields. In the medical field, their unique properties make them valuable assets. These materials exhibit healing properties and anticancer effects, rendering them valuable for drug delivery and targeted therapies. Additionally, their cytocompatible nature, along with antimicrobial, antifungal, and antiviral attributes, positions them as ideal candidates for wound and burn dressings, providing moisture retention, a gentle texture, and noninvasive monitoring capabilities.

Beyond the medical field, cellulose-based metallogels are finding their way into active food packaging, where they effectively control moisture and water activity, provide antibacterial protection, and act as food quality indicators by changing color at different pH levels.

In addition, these materials induce dye degradation and heavy metal absorption, contributing to efficient and safe water treatment processes. Magnetic-responsive metallogels have shown excellent performance in removing heavy metals from wastewater. The functional groups of the cellulose serve as active sites for metal ion retention, with the magnetic properties enhancing their adsorption capacity and recovery performance through the application of an external magnetic field.

Finally, metallogels have proven themselves valuable in catalytic processes, offering environmentally friendly and economically viable solutions for organic synthesis reactions, such as the reduction of 4-nitrophenol, Suzuki–Miyaura coupling reactions, and reactions between aromatic aldehydes and alcohols to produce aromatic acetals. Their ability to be recycled over multiple cycles makes them attractive as sustainable catalysts.

## 4. Discussion

The analysis of the total life cycle of cellulose-based metallogels in the three parts of the review “Cellulose-Based Metallogels” [12,21] allows us to suggest the possible challenges for the industrial production and application of these promising materials.

As shown in the first part of the review [21], one of the main challenges is the cost of production. The synthesis of cellulose hydrogels sometimes involves expensive solvent systems (DMAc/LiCl, NMMO, and DMSO/LiCl) and processing and post-processing steps (cross-linking, cooling, and freeze-drying for NaOH/aqueous systems). This may limit the scalability and commercial viability of these materials. In addition, the use of certain solvents (e.g., DMAc/LiCl) may have an environmental impact.

Finding green, environmentally friendly, and sustainable synthesis methods is critical to reducing the carbon footprint of these materials. Achieving precise control over the dispersion and size of metal nanoparticles within the cellulose matrix can be challenging. The development of efficient modification methods to ensure the uniform distribution and stability of metal nanoparticles is essential for consistent material performance. While cellulose is generally biocompatible, the introduction of metal nanoparticles can raise biocompatibility concerns, particularly in medical and pharmaceutical applications. Ensuring that these composites are safe for use in contact with biological systems is critical. Tailoring the surface properties of cellulose/metal nanoparticle composites for specific applications, such as selective adsorption or targeted drug delivery, can be complex. This is now being addressed using a variety of additives that introduce new functional groups. Overcoming these challenges will be essential to realize the full potential of cellulose/metal nanoparticle composites in various applications and industries.

## Data Availability

Not applicable.
